# *Portulaca oleracea* L. Polysaccharide Alleviates AFB_1_-Induced Liver Injury in New Zealand Rabbits via the Gut–Liver Axis

**DOI:** 10.3390/biology15151311

**Published:** 2026-08-05

**Authors:** Chunxiao Liu, Ruyi Hu, Yuqing Meng, Xiaoyan Niu, Liang Cao, Yuanqing Zhang

**Affiliations:** Shanxi Key Laboratory of Animal Nutrition and Feed Development, College of Animal Science, Shanxi Agricultural University, Jinzhong 030801, China; 240093@sxau.edu.cn (C.L.); nxysxau2022@163vip.com (X.N.)

**Keywords:** *Portulaca oleracea* L. polysaccharides, AFB_1_, liver injury, gut microbiota, New Zealand rabbit

## Abstract

Aflatoxin B_1_ (AFB_1_) is a prevalent mycotoxin contaminating food and environmental matrices that can induce hepatic injury, thereby posing a substantial threat to livestock production. *Portulaca oleracea* L. polysaccharide (POP) possesses diverse biological activities, including antioxidant and anti-inflammatory properties. However, the protective effects of POP against AFB_1_-induced hepatic injury have not been fully elucidated. In the present study, a rabbit model of AFB_1_-induced hepatic injury was established to investigate the hepatoprotective mechanisms of POP. Histopathological examination and liver function assessments demonstrated that POP markedly ameliorated hepatic histopathological alterations and decreased serum levels of alanine aminotransferase (ALT), aspartate aminotransferase (AST), and alkaline phosphatase (ALP). Molecular analyses further demonstrated that POP attenuated AFB_1_-induced oxidative stress and hepatocyte apoptosis, effects that were associated with activation of the PI3K/AKT signaling pathway and suppression of the mitochondria-mediated apoptotic pathway. Furthermore, microbiome profiling and targeted short-chain fatty acid (SCFA) metabolomics analyses indicated that POP supplementation contributed to the preservation of intestinal barrier integrity and intestinal microbiota stability, concurrent with the attenuation of AFB_1_-induced intestinal and hepatic injury and elevated SCFA production by intestinal microbes. Overall, POP exerts prominent preventive protective effects against AFB_1_-induced liver injury, which may be closely associated with alterations in intestinal microbiota composition and microbial short-chain fatty acid metabolism, as well as the activation of the PI3K/AKT signaling pathway. These findings provide novel insights into the mechanisms underlying AFB_1_-induced hepatic injury.

## Simple Summary

Aflatoxin B_1_ is frequently contaminated in animal feed, posing a significant threat to the rabbit industry. This study aims to evaluate the efficacy of adding *Portulaca oleracea* L. polysaccharides to the basic diet of New Zealand rabbits to alleviate the adverse effects of aflatoxin B. The results showed that *Portulaca oleracea* L. polysaccharides significantly reduced the hepatotoxicity induced by aflatoxin B_1_, restored the disrupted gut microbiota, and increased the content of short-chain fatty acids in the cecal contents. These results indicate that purslane polysaccharides have broad application prospects as a potential effective agent for degrading aflatoxin B1 in the feed industry.

## 1. Introduction

Aflatoxins represent a category of secondary toxic metabolites synthesized by Aspergillus flavus and Aspergillus parasiticus, commonly found in animal feedstuffs. AFB_1_ is the primary form of aflatoxin and ranks among the most prevalent and potent mycotoxins [1]. In animal production, feeding livestock with feed contaminated by AFB_1_ leads to organ damage, immunosuppression, increased susceptibility to disease, and reduced growth and reproductive performance, resulting in substantial losses for the farming industry [2]. Furthermore, AFB_1_ can be transmitted from contaminated feeds to livestock and poultry products, including meat, eggs, and milk. This issue also poses potential health risks to humans [3]. Consequently, there is an imperative necessity to ascertain efficient strategies for mitigating AFB_1_ pollution, thereby reducing the risks to both animals and humans.

The liver is the target organ for AFB_1_ exposure. Extensive research indicates that AFB_1_ exposure induces hepatic inflammation, connective tissue proliferation, and liver fibrosis [4]. AFB_1_ induces oxidative stress and apoptosis by stimulating excessive reactive oxygen species (ROS) production, ultimately leading to liver dysfunction [5,6]. Following AFB_1_ exposure, excessive ROS also inhibits the PI3K/AKT signaling pathway [5]. Apoptosis is an autonomous mechanism through which organisms remove damaged cells, thereby preserving internal homeostasis and promoting growth and development [7]. Mitochondria are essential for regulating hepatocyte apoptosis and are pivotal in energy production and reactive oxygen species (ROS) generation [8]. AFB_1_-induced oxidative stress and pro-inflammatory factors significantly increase, resulting in a decrease in mitochondrial outer membrane potential and the subsequent release of cytochrome c (cyt-c) into the cytoplasm [2]. This process initiates the caspase cascade and activates the mitochondrial-dependent apoptosis pathway, consequently worsening hepatic injury [2,9]. Consequently, the mitochondrial apoptosis signaling pathway represents the primary mechanism for AFB_1_-induced hepatocyte apoptosis in rabbits [2,10].

Numerous studies demonstrate that gut microbiota significantly influences liver immunity and disease [11]. It engages in host gut–liver communication and physiological regulation of organs by consistently providing numerous micronutrients and functional metabolites [12]. Research has revealed that AFB_1_ can impair gut microbial function by disrupting intestinal architecture, the intestinal barrier, and normal gut microbial communities in animals [13]. AFB_1_ exposure significantly decreased intestinal villus height and intestinal epithelial cell proliferation, while inhibiting the expression of tight junction proteins [14]. Recent research demonstrates that Pleurotus eryngii polysaccharides reduce AFB_1_-induced liver inflammation in ducks through the modulation of gut microbiota [3]. Salvia miltiorrhiza polysaccharides modified AFB_1_-induced gut microbiota dysbiosis and jejunal injury [7]. These results suggest that gut microbiota is essential for polysaccharide-mediated protection against AFB_1_-induced liver injury.

*Portulaca oleracea* L. (PO.L) is a food-medicine dual-use plant that has garnered significant attention in recent years for its anti-inflammatory, antioxidant, immunomodulatory, and antitumor activities [15]. Research indicates that PO.L can mitigate cadmium-induced damage to the liver, kidneys, and intestines [16]. Notably, water extracts of PO.L have been reported to inhibit AFB_1_ formation, achieving an inhibition rate of up to 81.7% [17]. Among the numerous bioactive components of PO.L, polysaccharides are particularly significant as multi-functional biological response modifiers [18]. POP has been documented to reduce hepatic oxidative stress in diabetic rats and alleviate ulcerative colitis by regulating gut microbial homeostasis [19]. Based on this, POP may represent a potential natural functional food for alleviating aflatoxin poisoning. However, the mechanism by which POP mitigates AFB_1_-induced liver and intestinal damage in rabbits remains unclear.

This study investigated that POP may exert preventive protective effects against AFB_1_-induced toxicity and explored the underlying mechanisms by assessing hepatic oxidative stress, apoptosis, intestinal barrier damage, gut microbiota, and SCFA metabolites. This offers a theoretical framework for investigating POP as a therapeutic agent to alleviate AFB_1_-induced liver injury.

## 2. Materials and Methods

### 2.1. Animals and Experimental Design

Thirty 35-day-old weaned New Zealand rabbits with an initial body weight of 700 ± 50 g were enrolled in this study. All experimental rabbits were first stratified by gender and equally allocated, then randomly assigned into five subgroups (*n* = 6 per group, with equal numbers of male and female rabbits). The rabbit model of AFB1-induced liver injury was established using the previously established method [20]. To construct a preventive intervention model against AFB_1_-induced toxicity, a 21-day continuous POP pre-administration regimen was applied prior to AFB_1_ challenge in the combination groups. The control group (Ctrl) was given free access to water and olive oil; the AFB_1_ group was given 0.3 mg/kg AFB_1_ daily for the last 5 days of the experiment (Qingdao Pribo Laboratory Co., Ltd., Qingdao, China); the single POP group was given 400 mg/kg POP continuously for 21 days; the AFB_1_ + POP(H) and AFB_1_ + POP(L) groups were given 400 mg/kg and 200 mg/kg POP respectively for 21 days, followed by continuous oral administration of 0.3 mg/kg AFB_1_ for the last 5 days. AFB_1_ dissolved in olive oil. At the conclusion of the experiment, body weight and liver weight were measured; serum, liver, jejunal, and cecal contents were collected and stored at −80 °C.

### 2.2. Preparation of Portulaca oleracea L. Polysaccharides

The *Portulaca oleracea* L. used in this experiment was collected in Binzhou (Shandong, China) in August 2024 and was identified as *Portulaca oleracea* L. by Professor Weiling Wang, a botany professor from Shanxi Normal University.

The fresh *Portulaca oleracea* L. was washed clean, dried, and then placed in a drying oven to be dried and ground. The extraction of polysaccharides was improved based on the previous methods [21]. The *Portulaca oleracea* L. powder was soaked in petroleum ether in a 2:1 ratio overnight, then filtered using nylon cloth to remove fat and some pigments, and then soaked in anhydrous ethanol to remove the remaining pigments. It was then dried at room temperature, and extracted three times with boiling distilled water for 2 h each. The supernatant was filtered through 16 layers of gauze, and centrifuged. The obtained supernatant was mixed and concentrated to one-tenth of its original volume using a rotary evaporator at approximately 50 °C. The concentrated solution was added to four times its volume of 95% ethanol and left to overnight at 4 °C for alcohol precipitation. The precipitate was centrifuged, washed with anhydrous ethanol, dissolved in distilled water, and centrifuged to remove the grayish-white precipitate, and then freeze-dried to obtain crude polysaccharide powder. The crude polysaccharide was dissolved in water at a 1:100 ratio, and then mixed with papain and incubated at 60 °C for 4 h in a water bath to digest proteins. After the digestion was completed, it was incubated at 100 °C for 10 min. The concentrated solution was rotary evaporated to a certain volume and added to an equal volume of Sevage (V chloroform: V n-butanol = 4:1) reagent for ultrasonic oscillation to remove proteins. The solution was left to precipitate at 4 °C with anhydrous ethanol overnight. Then, it was subjected to 3500 Da dialysis bag for separation and finally freeze-dried to obtain polysaccharide POP. The total sugar content was measured using the phenol–sulfuric acid method and was found to be 65.78%.

### 2.3. Monosaccharide Composition

Monosaccharide composition was determined by high-performance liquid chromatography (HPLC; LC-20AT, Shimadzu, Kyoto, Japan) after pre-column derivatisation with 1-phenyl-3-methyl-5-pyrazolone. Briefly, POP were hydrolysed with trifluoroacetic acid at 120 °C, neutralised, derivatised with PMP, and analysed on a C18 column at 245 nm.

### 2.4. FT-IR

The structural characteristics of POP were analyzed using the Nicolet iS50 spectrophotometer (Thermo Fisher Scientific, Wilmington, DE, USA) through Fourier transform infrared spectroscopy. The spectral scans were conducted within the wavenumber range of 4000–400 cm^−1^ with a resolution of 4 cm^−1^. During sample preparation, the purified POP powder was thoroughly mixed with spectral-grade potassium bromide, and the homogeneous mixture was pressed into particles for spectral detection.

### 2.5. Determination of Serum Biochemical Indicators

Blood was collected from rabbit hearts, and serum was prepared by centrifuging at 3500 g for 10 min. Serum liver function markers aspartate transaminase (AST), alanine transaminase (ALT), and alkaline phosphatase (ALP) were measured utilizing commercial kits (Nanjing JianCheng Bioengineering Institute, Nanjing, China).

### 2.6. Liver Oxidative Stress Markers Assay

Liver tissue was homogenized using a homogenizer and mixed with nine volumes of physiological saline at 4 °C, after which the supernatant was collected. Concentrations of oxidative stress indicators T-SOD, MDA, and GSH-PX were measured utilizing a kit in accordance with the manufacturer’s guidelines (Servicebio Technology Co., Ltd., Wuhan, China). Additionally, reactive oxygen species (ROS) were identified with a dihydroethidium (DHE) fluorescent probe test in a fluorescence microplate scanner.

### 2.7. Histopathological Assessment

Fresh liver and intestinal tissues were rinsed with phosphate-buffered saline (PBS) and subsequently fixed in 4% paraformaldehyde for 48 h. Following fixation, tissues were rinsed thoroughly, dehydrated through a graded ethanol series, and embedded in paraffin wax. Serial sections of 5 μm thickness were prepared, stained with hematoxylin and eosin (H&E), and examined using a light microscope to assess tissue morphology.

### 2.8. Apoptosis Assay by TUNEL

Liver tissue was embedded and sectioned at 4 μm thickness, then dewaxed in xylene, treated with proteinase K, and decolored with PBS. Apoptosis in the liver was assessed utilizing a TUNEL assay kit, following the manufacturer’s guidelines (Servicebio Technology Co., Ltd., Wuhan, China). In brief, sections were incubated with an appropriate amount of TDT enzyme and dUTP at 37 °C for 2 h. Following subsequent DAPI counterstaining of nuclei, sections were examined under a fluorescence microscope and photographed. Nuclei of positively apoptotic cells appeared green, and the apoptosis rate was calculated using ImageJ software (Version 1.52v. National Institutes of Heealth, Bethesda, BD, USA).

### 2.9. Quantitative Real-Time PCR

Total RNA was isolated from the jejunum and liver using Trizol reagent (CoWin Biotechnology Co., Ltd., Beijing, China) and analyzed as previously described. Total RNA underwent reverse transcription to cDNA utilizing the PrimeScript RT Reagent Kit (TaKaRa Bio Inc., Kusatsu, Japan). Real-time quantitative PCR was conducted utilizing the CFX96 PCR system (Bio-Rad Laboratories, Inc., Hercules, CA, USA) and MagicSYBR Mixture (CoWin Biotechnology Co., Ltd., Beijing, China) in accordance with the manufacturer’s guidelines. Primers for quantitative polymerase chain reaction (PCR) were synthesized by Sangon Biotech Co., Ltd. in Shanghai, China (Supplementary Table S1). Gene expression levels were analyzed utilizing the 2^−ΔΔCT^ method.

### 2.10. Western Blotting Assay

Grind liver tissue in liquid nitrogen using a grinder. Extract total protein using RIPA protein lysis buffer containing the protease inhibitor phenylmethylsulfonyl fluoride (PMSF) (Servicebio Technology Co., Ltd., Wuhan, China), and quantify using the BCA protein assay kit (Thermo Scientific, Waltham, MA, USA). Mix with SDS sample buffer prior to boiling, then separate 20–30 μg of protein on a polyacrylamide gel and transfer to a polyvinylidene fluoride (PVDF) membrane. Incubate the membrane overnight at 4 °C with primary antibodies followed by incubation with corresponding secondary antibodies at room temperature for 2 h. Following visualization and photographing of protein bands, quantitative expression analysis was performed using ImageJ software (USA).

### 2.11. 16S rRNA Sequencing

DNA was extracted from cecal contents using the MagPure Soil DNA LQ Kit (Magan Biotechnology Co., Ltd., Guangzhou, China) according to the manufacturer’s protocol. DNA quality was assessed via 1% agarose gel electrophoresis, whilst concentration and purity were determined using a NanoDrop2000 instrument (Thermo Fisher Scientific, Wilmington, DE, USA). PCR amplification targeted the V3-V4 region of the 16S rRNA gene. Sequencing was performed using the Illumina NovaSeq 6000 platform (OE Biotechnology Co., Ltd., Shanghai, China) with 2 × 250 bp paired-end reads. Based on ASV and abundance data, QIIME 2 software was employed to calculate α-diversity indices and β-diversity distance matrices. Significance of species abundance differences was assessed using ANOVA/Kruskal–Wallis/*t*-tests/Wilcoxon tests within the R package (R version 3.6.0). Linear discriminant analysis effect size (LEfSe) was applied to screen differentially abundant microbial taxa. False discovery rate (FDR) correction was performed for multiple testing in taxonomic difference analyses. The SILVA database (https://ftp.arb-silva.de, accessed on 31 December 2024) was utilized as the reference database for microbial taxonomic annotation.

### 2.12. LC-MS Analysis of SCFAs

Collected cecal contents samples from domestic rabbits were rapidly frozen in liquid nitrogen and stored at −80 °C. Short-chain fatty acids (SCFAs) were measured using ultra-high-performance liquid chromatography–mass spectrometry (UHPLC-MS/MS) sys (Waters Corporation, Milford, CT, USA; AB Sxiex LLC, Marlboroough, MA, USA). Metabolite quantification was performed using multiple reaction monitoring (MRM) mode on a triple quadrupole mass spectrometer.

### 2.13. Network Pharmacology Analysis

Potential targets for *Portulaca oleracea* L. were collected via the Traditional Chinese Medicine Systems Pharmacology (TCMSP) system (https://www.tcmsp-e.com/#/database, accessed on 12 November 2024), with criteria of oral bioavailability ≥30% and drug-likeness ≥0.18. Using ‘Aflatoxin B1 induced liver injury’ as the search keyword, relevant genes were collected from Genecards (relevance score > 5). An online tool (https://bioinformatics.com.cn/, accessed on 17 February 2025) was employed to generate a Venn diagram, where the intersection represents potential targets for POP mitigation of AFB_1_-induced liver injury. The obtained intersection targets were analyzed using the STRING database. Subsequently, the protein–protein interaction (PPI) network diagram was imported into Cytoscape software (Version 3.8.0. CA, USA) to screen core genes and construct a relevant core protein network. To elucidate the roles of POP target genes in alleviating AFB_1_-induced liver injury within gene functions and signaling pathways, we performed Gene Ontology (GO) and KEGG pathway enrichment analyses on the potential targets using the DAVID database.

### 2.14. Statistics and Analysis

Statistical analyses were conducted utilizing GraphPad Prism 8.0 software (USA). Each independent experiment was conducted three times. Data are presented as Mean ± SEM. Comparisons between two groups utilized *t*-tests, whereas comparisons among multiple groups employed one-way analysis of variance (ANOVA). * or # *p* < 0.05, ** or ## *p* < 0.01, and “ns” indicates no significant difference.

## 3. Results

### 3.1. POP Prevented AFB_1_-Induced Liver Injury in Rabbits

The POP identified through HPLC is composed of galactose, galacturonic acid, arabinose, rhamnose, glucose, glucuronic acid, and mannose, with molar percentages of 35.8%, 22.1%, 20.8%, 11.2%, 5.7%, 2.5%, and 1.84%, respectively (Supplementary Figure S1). The typical and characteristic absorption peaks of polysaccharides were observed in the range of 4000–400 cm^−1^. The broad and strong absorption band at approximately 3400 cm^−1^ corresponded to the stretching vibration of hydroxyl (-OH) groups in polysaccharide chains. The peak near 2900 cm^−1^ was attributed to C-H stretching vibration of aliphatic hydrocarbon groups. The characteristic absorption peaks at 1000–1200 cm^−1^ represented the stretching vibration of C-O-C and C-OH, which are the typical fingerprint features of pyranose polysaccharides (Supplementary Figure S2).

**Figure 1:**
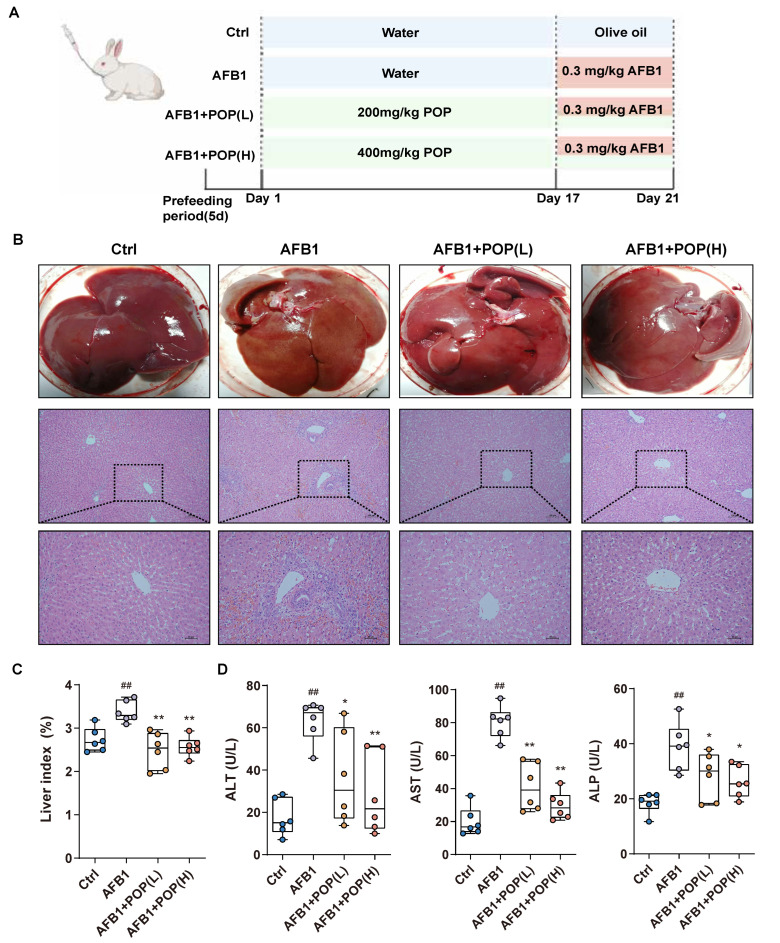
POP alleviates AFB1-induced liver injury in rabbits. (**A**) Animal experimental design diagram. (**B**) Representative H&E-stained images of rabbit liver (scale bars = 100 μm and 50 μm). (**C**) Liver index. (**D**) Serum ALT, AST, and ALP levels. Mean ± SEM (*n* = 6), each dot represents an individual rabbit. ## Compared with control group. * Compared with AFB1 group. * *p* < 0.05, ## or ** *p* < 0.01.

Following dissection of rabbit livers, the control group exhibited a smooth hepatic capsule with a reddish-brown coloration. Hepatocytes displayed normal morphology, orderly arrangement, and intact structure. In contrast, the AFB_1_ group demonstrated marked abnormalities, including a pale yellow hepatic coloration and a fragile texture. Further histopathological examination revealed disordered hepatocyte arrangement, partial dissolution and fragmentation of cell nuclei, alongside inflammatory cell infiltration and interstitial hemorrhage. Following POP treatment, the liver exhibited a reddish-brown appearance with orderly hepatocyte arrangement, and a marked reduction in inflammatory cell and hemorrhagic infiltration. POP significantly alleviated hepatic morphological abnormalities and histopathological damage (Figure 1B). Compared to the control group, the AFB_1_ group exhibited significantly elevated hepatic indices, whereas POP groups markedly reduced AFB_1_-induced increases in these indices (Figure 1C). To further characterize hepatic injury, serum levels of ALT, AST, and ALP were measured. Serum levels of ALT, AST, and ALP in the AFB_1_ group were considerably higher compared to the control (Figure 1D). Compared with the AFB_1_ rabbit, POP significantly reversed the elevation in these liver function indicators (Figure 1D). POP significantly alleviated AFB_1_-induced morphological alterations in liver tissue and liver function impairment. In addition, normal rabbits were fed with 400 mg/kg POP to evaluate the safety of this dosage. Compared with the control group, rabbits administered with POP presented normal hepatocyte morphology and intact hepatic structure, with no significant differences in hepatic organ indices as well as serum ALT and AST levels (Supplementary Figure S3).

### 3.2. Network Pharmacology Predicts Potential Targets

**Figure 2:**
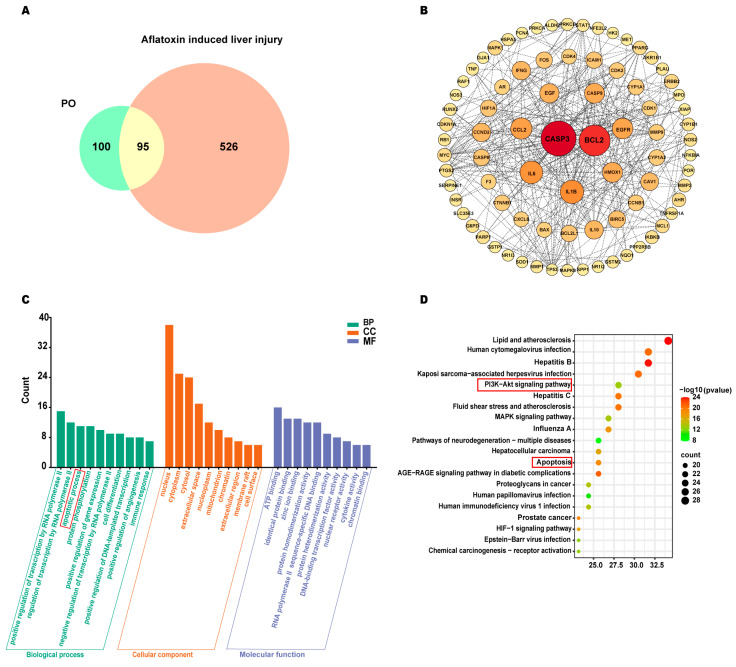
Potential targets of POP against AFB1-induced liver injury. (**A**) Common target genes of *Portulaca oleracea* L. and AFB1-induced liver injury. (**B**) PPI network analysis of core genes. (**C**) GO enrichment analysis. (**D**) KEGG enrichment analysis.

Network pharmacology analysis was performed to identify the potential targets of POP against AFB_1_-induced hepatic injury. A total of 195 potential target genes for purslane were obtained from the TCMSP database, while 626 target genes associated with ‘AFB_1_-induced liver injury’ were retrieved from the GeneCards database. Ultimately, 95 intersecting genes were identified as potential targets for mitigating AFB_1_-induced liver injury (Figure 2A). Concurrently, the STRING website generated a protein–protein interaction (PPI) network, and the identified core genes were further analyzed using Cytoscape (Figure 2B). Among the top 10 core targets, Caspase 3, Bcl2, Caspase 9, EGFR, and EGF were closely associated with apoptosis [2], while IL6, IL1β, IL10, and C-CL2 were strongly linked to inflammation [22]. HOMX1 participated in oxidative stress [23]. Subsequently, GO and KEGG enrichment analyses were performed on 95 intersecting target genes from purslane and AFB_1_-induced liver injury (Figure 2B,C). The enriched BP terms participated in the apoptosis process (Figure 2C). Among the top 20 KEGG pathways enriched, the PI3K/Akt, MAPK, and apoptosis signaling pathways were closely associated with AFB_1_-induced liver injury (Figure 2D), suggesting that POP may alleviate AFB_1_-induced toxic liver injury by influencing these biological functions.

### 3.3. POP Inhibits AFB_1_-Induced ROS Production and Activates the PI3K/AKT Signaling Pathway in Rabbit Liver

**Figure 3:**
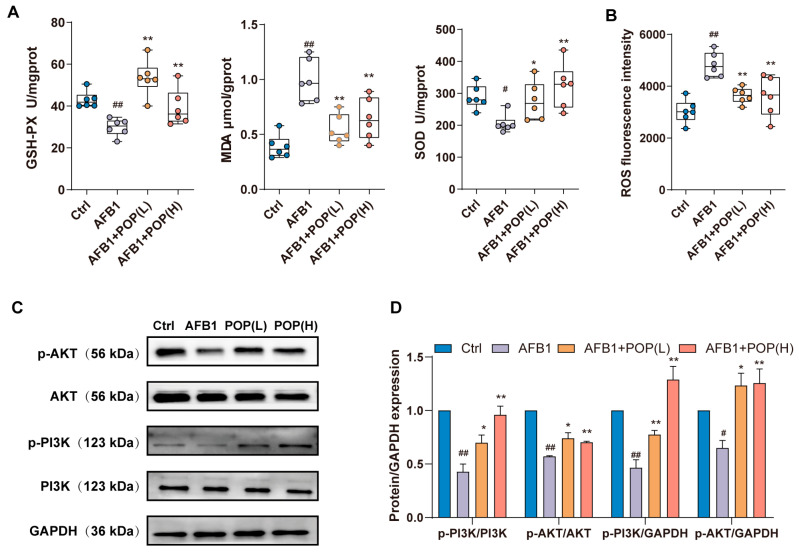
Effects of POP on AFB1-induced oxidative stress in rabbit liver. (**A**) Levels of oxidative stress markers SOD, MDA and GSH-px in the liver (*n* = 6), each dot represents an individual rabbit. (**B**) Fluorescence intensity of ROS. (**C**) Western blot analysis of protein expression levels for PI3K, p-PI3K, AKT, and p-AKT. (**D**) Band quantification and statistical analysis using ImageJ software. # Compared with control group; * Compared with AFB1 group. # or * *p* < 0.05, ## or ** *p* < 0.01.

To verify whether POP alleviates AFB_1_-induced oxidative stress, we assessed oxidative stress levels in rabbit liver tissue. Compared with the control group, AFB_1_ significantly induced ROS and MDA levels while conversely reducing SOD and GSH-PX levels (Figure 3A,B). Treatment with both doses of POP significantly reversed AFB_1_-induced oxidative stress (Figure 3A,B). ROS can modulate the PI3K/AKT signaling pathway through multiple mechanisms [24]. Consequently, Western blot analysis of PI3K/AKT pathway expression revealed that AFB_1_ exposure suppressed PI3K and Akt protein phosphorylation levels in rabbit liver tissue compared to controls. In contrast, POP treatment significantly increased the protein expression levels of p-PI3K, p-AKT, p-PI3K/PI3K and p-Akt/Akt (Figure 3C,D). These findings suggest that POP may alleviate AFB_1_-induced oxidative stress by inhibiting ROS production and activating the phosphorylation of the PI3K/AKT signaling pathway.

### 3.4. POP Alleviates AFB_1_-Induced Mitochondrial-Dependent Apoptosis in Rabbit Liver

**Figure 4:**
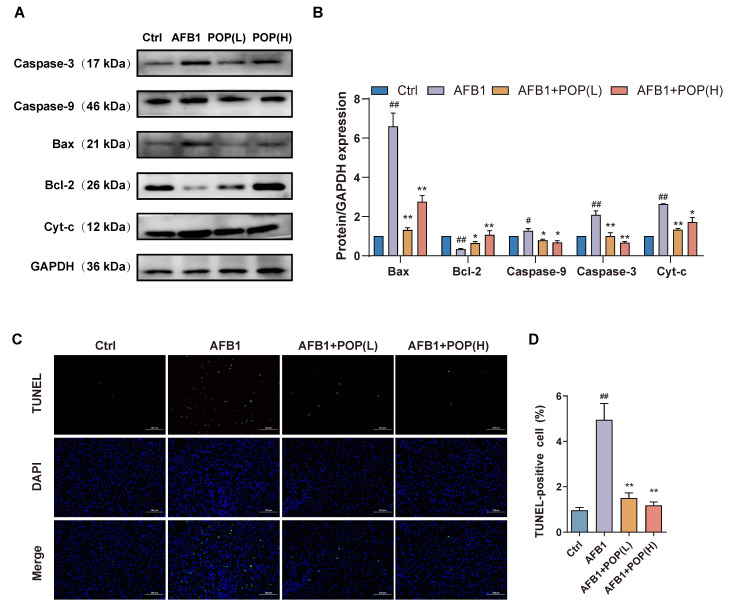
Effects of POP on AFB1-induced mitochondrial-dependent apoptosis in rabbit liver. (**A**) Western blot analysis of key mitochondrial apoptosis pathway proteins Caspase 3, Caspase 9, Bax, Bcl-2, and cyt-c in the liver. (**B**) Quantitative analysis of bands using ImageJ software. (**C**) Representative TUNEL staining images of apoptotic cells in rabbit liver. (**D**) Statistical analysis of apoptotic cell rate using ImageJ. # Compared with control group; * Compared with AFB1 group. # or * *p* < 0.05, ## or ** *p* < 0.01.

AFB_1_ induces oxidative stress and activates the mitochondrial-dependent apoptotic pathway, ultimately leading to hepatocyte apoptosis. Western blot analysis revealed that compared to the control group, AFB_1_ increased the expression of caspase-3, caspase-9, Bax, and cyt-c proteins while decreasing Bcl2 protein expression. However, POP groups reversed the expression of crucial proteins in the AFB_1_-induced mitochondrial apoptosis signaling pathway (Figure 4A,B). Furthermore, the number of TUNEL-positive cells in rabbit livers from the AFB_1_ group was significantly higher than in the control group, whereas the POP-treated group exhibited reduced AFB_1_-induced hepatocyte apoptosis (Figure 4C,D). These findings suggest that POP may alleviate AFB_1_-induced apoptosis by modulating the mitochondrial-dependent apoptotic pathway.

### 3.5. POP Ameliorates AFB_1_-Induced Intestinal Barrier Damage in Rabbits

**Figure 5:**
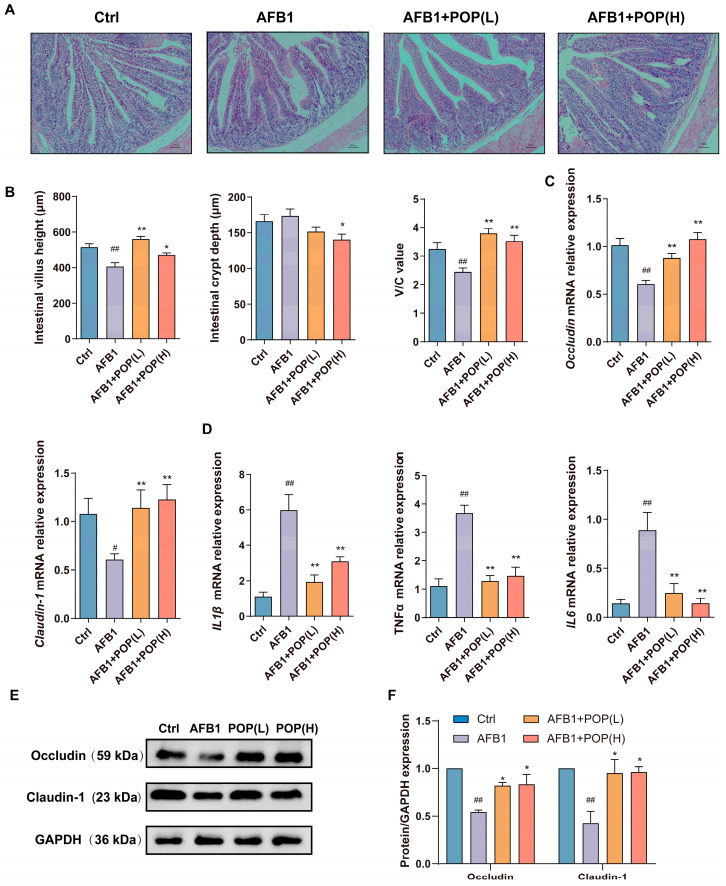
Effects of POP on AFB1-induced intestinal barrier damage. (**A**) Representative H&E-stained image of the jejunum (scale bar = 100 μm). (**B**) ImageJ software measured villus height and crypt depth, analyzing the villus-to-crypt ratio. (**C**) qRT-PCR detection of mRNA expression levels for intestinal tight junction proteins *Occludin* and *claudin-1*. (**D**) qRT-PCR detection of mRNA expression levels for inflammatory factor-related genes *IL-6*, *IL-1β*, and *TNFα*. (**E**) Western blot analysis of protein expression levels for Occludin, Claudin-1. (**F**) Quantitative analysis of bands using ImageJ software.# Compared with control group; * Compared with AFB1 group. # or * *p* < 0.05, ## or ** *p* < 0.01.

To investigate the effect of POP on intestinal barrier integrity in rabbits treated with AFB_1_, we analyzed the intestinal barrier via villus morphology assessment (Figure 5A). Compared with the control, the AFB_1_ group exhibited reduced villus height and a significantly decreased villus-to-crypt ratio (Figure 5B). We further observed decreased *occludin* and *claudin-1* mRNA levels in jejunal tissue from the AFB_1_ group (Figure 5C), alongside elevated *IL-1β*, *IL-6* and *TNFα* mRNA expression (Figure 5D). POP treatment significantly increased villus height, V/C ratio, and mRNA levels of *occludin* and *claudin-1* in AFB_1_ rabbits, whilst reducing inflammatory factor expression (Figure 5B–D). Western blot analysis revealed that AFB1 exposure markedly downregulated the protein expression of occludin and claudin-1. Conversely, POP treatment effectively restored the AFB1-induced reduction in the expression of these tight junction proteins (Figure 5E,F). This suggests POP ameliorates AFB_1_-induced intestinal barrier damage in rabbits.

### 3.6. POP Restores AFB_1_-Induced Gut Microbiota Imbalance in Rabbits

**Figure 6:**
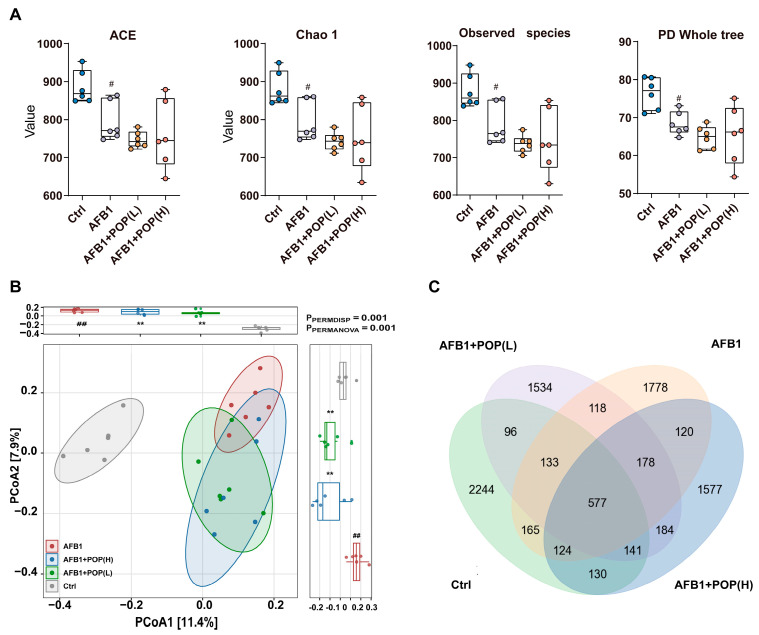
Effects of POP and AFB_1_ on gut microbiota diversity. (**A**) α-diversity indices: ACE, Chao 1, Observed species, and PD. (**B**) Plot-based UniFrac PCoA. (**C**) Venn diagram at the ASV level. (*n* = 6) each dot represents an individual rabbit. # control compared with AFB_1_ group; ** POP compared with AFB_1_ group. # *p* < 0.05, ## or ** *p* < 0.01.

Increasing evidence suggests that AFB_1_ exposure is closely associated with the gut microbiota [20]. Therefore, we used systematic sequencing of 16S rRNA gene amplicons to analyze the composition of the gut microbiota in rabbit cecal contents. Compared with the control, AFB_1_ reduced species richness indices, including ACE, Chao1, observed species, and PD (Figure 6A), indicating that AFB_1_ diminished the species richness and diversity of gut microbiota. POP treatment failed to restore gut microbial diversity and richness. However, PCoA analysis of β-diversity revealed that the gut microbiota structure in the AFB_1_ group was markedly segregated from other groups (Figure 6B). Furthermore, the petal plot revealed that different interventions influenced the gut microbiota (Figure 6C). These findings collectively indicate that POP can alter the structure of the gut microbiota in rabbits.

**Figure 7:**
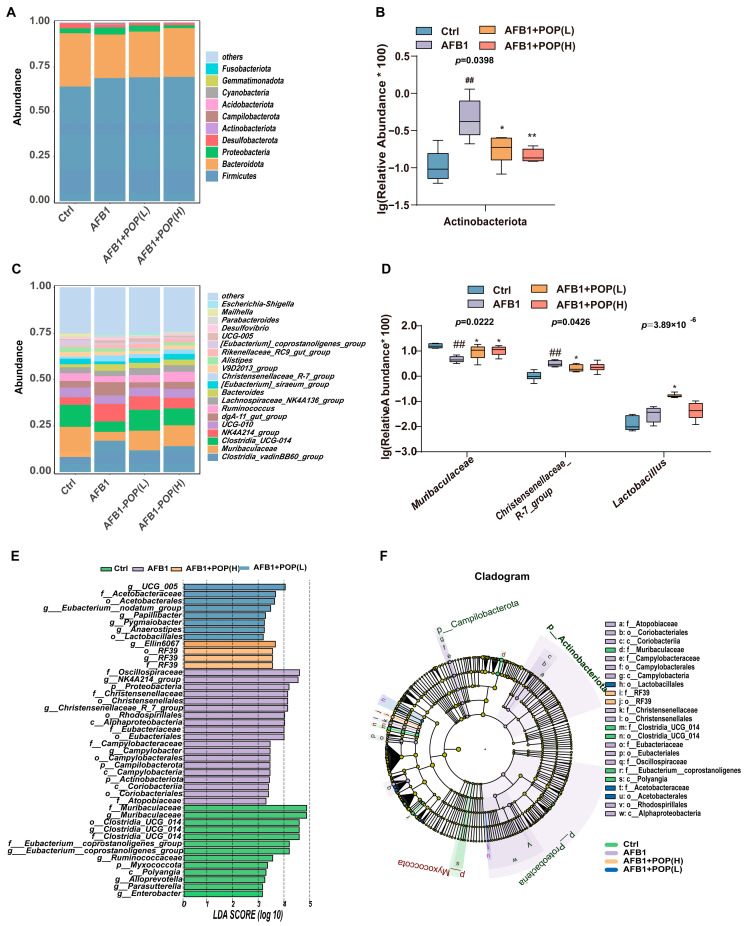
Effects of POP and AFB_1_ on gut microbiota composition. (**A**) Gut microbiota composition at the phylum level (top 10). (**B**) Significant differences in gut microbiota composition at the phylum level. (**C**) Gut microbiota composition at the genus level (top 20). (**D**) Significant differences in gut microbiota composition at the genus level. (**E**) Histogram of LDA score distribution. (**F**) Evolutionary branch diagram from LEfSe analysis. mean ± SEM (*n* = 6). ## control compared with AFB_1_ group; * POP compared with AFB_1_ group. * *p* < 0.01, ## or ** *p* < 0.01.

We assessed the relative abundance of major bacterial taxa to analyze compositional changes in the gut microbiota. At the phylum level (top 10), the AFB_1_ group exhibited increased relative abundance of Actinobacteriota compared to the control group. POP treatment reversed the AFB_1_-induced relative abundance of these two bacterial phyla (Figure 7A,B). At the genus level, the relative abundance of *Muribaculaceae* was significantly decreased in the AFB_1_ group compared with the control group, while that of *Christensenellaceae_R-7_group* was increased (Figure 7C,D). Concurrently, low-dose POP treatment restored the relative abundances of *Muribaculaceae* and *Lactobacillus* and reduced the abundance of *Christensenellaceae_R-7_group*. High-dose POP treatment also rescued the relative abundance of *Muribaculaceae* (Figure 7C,D). Furthermore, to screen potential bacterial biomarkers responsible for the protective effect of POP against AFB_1_-induced liver injury, we performed LEfSe analysis and cladogram visualization (Figure 7E). Obvious discrepancies in bacterial taxonomic composition were detected among different groups. Specifically, taxa including o__Lactobacillales, f__Acetobacteraceae, *g__Pygmaiobacter,* and *g__Anaerostipes* were enriched in the AFB_1_ + POP (L) group (Figure 7E,F), whereas *g__RF39* was markedly enriched in the AFB_1_ + POP (H) group (Figure 7E,F). Collectively, these results demonstrated that POP intervention effectively ameliorated AFB_1_-induced gut microbiota dysbiosis in rabbits.

### 3.7. POP-Regulated Microbiota-Derived SCFAs Transport via the Gut–Liver Axis in AFB_1_ Rabbit

**Figure 8:**
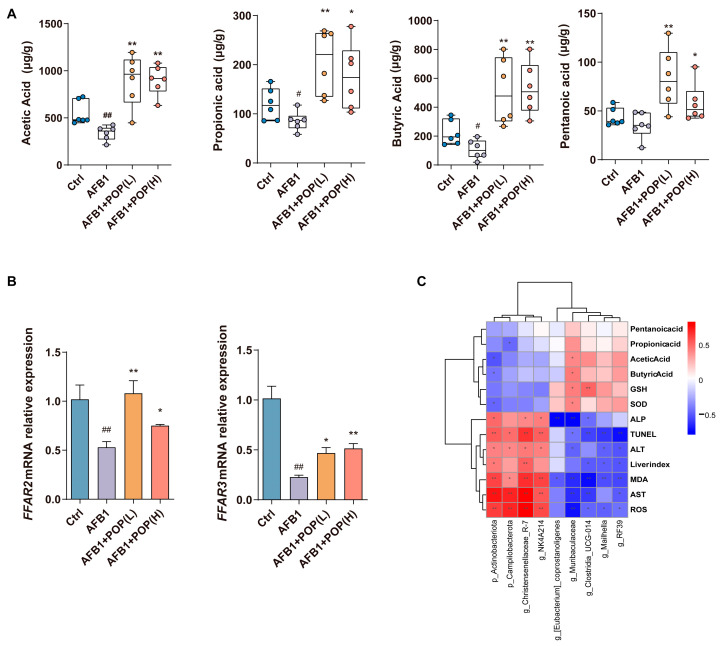
Effects of POP and AFB_1_ on rabbit intestinal short-chain fatty acids and their hepatic receptors. (**A**) Metabolomic analysis of acetic acid, propionic acid, butyric Acid, and pentanoic acid (μg/g) concentrations in caecal content (*n* = 6), each dot represents an individual rabbit. (**B**) qRT-PCR detection of SCFAs receptor *FFAR2* and *FFAR3* expression levels in rabbit liver. (**C**) Heatmap of liver-related indicators, gut microbiota, and SCFAs analyzed by Spearman correlation. # control compared with AFB_1_ group; * POP compared with AFB_1_ group. # or * *p* < 0.05, ## or ** *p* < 0.01.

As key microbiota-derived metabolites, SCFA production is influenced by the gut microbiota [3]. We further investigated the effects of POP on SCFA concentrations in cecal contents and on the expression of SCFA transport-related genes in the liver. Compared with the control, concentrations of acetic acid, propionic acid, and butyric acid were significantly reduced in AFB_1_ groups, whereas POP treatment significantly increased the concentrations of acetic acid, propionic acid, butyric acid, and pentanoic acid (Figure 8A). Concurrently, POP treatment markedly reversed the AFB_1_-induced reduction in *FFAR2* and *FFAR3* expression in rabbit liver (Figure 8B). Further Spearman correlation analysis was conducted to explore relationships between gut microbiota, SCFA concentrations in cecal contents, liver injury markers, and differentially abundant gut bacteria (Figure 8C). These data indicate that POP effectively elevated SCFA concentrations in the gut, alleviating the SCFA transport impairment induced by AFB_1_ via the gut–liver axis.

## 4. Discussion

AFB_1_ contamination represents a significant concern in global livestock farming, with increasingly severe detrimental effects on animal liver health [25]. Polysaccharides, as multi-functional biological response modifiers, mitigate the toxicity of aflatoxins in animals through multiple pathways [3,7]. POP, an essential component of *Portulaca oleracea* L., demonstrates various pharmacological activities, including anti-inflammatory and antioxidant effects [15]. Studies demonstrate that POP may alleviate acute liver injury through the enhancement of antioxidant enzymes and the reduction in oxidative stress [26]. Our research demonstrates that POP alleviates AFB_1_-induced liver injury in rabbits by activating the PI3K/AKT signaling pathway through reducing oxidative stress, subsequently modulating the mitochondrial apoptosis pathway to inhibit hepatocyte apoptosis. This process is accompanied by the regulation of the gut microbiota as well as a significant increase in SCFAs.

The liver is the primary organ involved in animal metabolism and also the organ most severely damaged following AFB_1_ exposure. Extensive research indicates that AFB_1_ exposure induces hepatic inflammation, connective tissue proliferation, hematoma formation, and liver fibrosis [20], alongside elevated serum levels of hepatic function markers, including ALT, AST, and ALP activity [27]. This indicates severe AFB_1_-induced hepatic dysfunction, consistent with our findings. In this study, our established AFB_1_-induced liver injury model revealed yellowing of rabbit livers following five consecutive days of 0.3 mg/kg/d AFB_1_ administration, alongside histological alterations including disrupted cellular arrangement, inflammatory infiltration, and hematoma formation. Concurrently, serum levels of hepatic function markers ALT, AST, and ALP significantly increased. Treatment with POP significantly mitigated AFB_1_-induced hepatic tissue structural damage and restored serum hepatic function marker levels. This indicates that POP administration can alleviate AFB_1_-induced hepatic injury.

AFB_1_ induces hepatic oxidative stress and apoptosis by stimulating excessive ROS production, ultimately leading to liver dysfunction [5,6]. In this study, AFB_1_ significantly elevated oxidative stress enzyme levels in rabbit liver, which were markedly reduced following POP administration. The PI3K/AKT signaling pathway is a crucial pathway involved in regulating cell growth, metabolism, and apoptosis. ROS can modulate PI3K/AKT signaling [28]. Lin et al. found that AFB_1_ exposure suppressed PI3K and AKT protein expression in chicken liver [5]. Consistent with our results. In this study, PI3K and AKT protein phosphorylation levels were significantly reduced in AFB_1_-treated livers, whereas POP administration markedly reactivated the PI3K/AKT signaling pathway. Furthermore, activated PI3K promotes downstream Akt activation, which subsequently counteracts AFB_1_-induced hepatocyte apoptosis by inhibiting Bax release and upregulating Bcl-2 [5,29]. Previous studies have indicated that the mitochondrial apoptosis signaling pathway constitutes the primary route for AFB_1_-induced hepatocyte apoptosis in rabbits [10]. Our results demonstrated that AFB_1_ induced alterations in the mitochondrial apoptosis pathway, specifically affecting the protein expression of Caspase 3, Caspase 9, cyt-c, and Bax within the mitochondrial apoptosis pathway, while suppressing Bcl-2 protein expression. Administration of POP restored the altered expression levels of these key proteins. This indicates that POP may activate the PI3K/Akt signaling pathway by inhibiting ROS production, subsequently blocking the mitochondrial apoptosis pathway and ultimately suppressing AFB_1_-induced apoptosis.

The gut–liver axis plays a crucial role in sustaining liver health [11]. Research indicates that gut microbiota play a significant role in AFB_1_-induced liver injury in both mice and broiler chickens [9,30]. AFB_1_ can impair gut microbial function by disrupting intestinal architecture, the intestinal barrier, and normal gut microbial communities [13]. This aligns with our findings, which revealed significantly reduced intestinal villus height, V/C ratio, and tight junction protein expression in AFB_1_-treated rabbits, alongside markedly elevated inflammatory cytokine expression. Conversely, POP treatment protected the gut from AFB_1_-induced barrier damage, increased tight junction protein expression, and reduced inflammatory cytokine expression. Previous studies have demonstrated that RBMX2 dysregulation during bacterial infection drives abnormal EMT activation and mediates excessive epithelial apoptosis, thereby aggravating mucosal barrier collapse and tissue injury [31]. In line with these findings, our results further confirm that POP intervention can alleviate AFB_1_-triggered tight junction protein loss, and inhibit excessive hepatocellular apoptosis, which collectively contributes to the improvement in intestinal and hepatic tissue damage.

Further microbiome analysis revealed that the relative abundance of Actinobacteriota and Campylobacterota significantly increased following AFB_1_ treatment, whereas POP downregulated the relative abundance of both phyla. Previous studies have documented a marked increase in relative Actinobacteriota abundance in fecal samples from AFB_1_-treated mice [32]. At the genus level, feeding POP significantly restored the AFB_1_-induced reduction in relative abundance of *Muribaculaceae*. Xu et al. also confirmed this, demonstrating that AFB_1_ treatment significantly decreased the relative abundance of *Muribaculaceae*, while administration of humic acids restored *Muribaculaceae* abundance and alleviated AFB_1_-induced liver injury [33]. *Muribaculaceae* can produce SCFAs [34]. The LEfSe phylogenetic cladogram demonstrated a significant enrichment of *Lactobacillales* in the POP-L treatment group, identifying this taxon as a key microbial biomarker. Previous studies have reported that members of *Lactobacillales*, particularly *Lactobacillus* species, are commonly incorporated into animal feed because of their capacity to biodegrade AFB1 [35]. These findings suggest that the detoxifying activity of POP may be associated with its ability to modulate the abundance of beneficial microbial taxa, including *Lactobacillales* and *Muribaculaceae*, thereby contributing to the degradation and removal of AFB1.

Short-chain fatty acids (SCFAs) are prominent microbial metabolites in the intestinal tract and mediate bidirectional interactions between the intestinal microecology and the host. Previous studies have confirmed that AFB_1_ exposure is negatively correlated with intestinal SCFA abundance [36]. By binding to and activating free fatty acid receptors (FFARs), SCFAs participate in the regulation of cellular apoptosis and pro-inflammatory cytokine production [37]. In the present study, Muribaculaceae and Lactobacillus, recognized as crucial SCFA-producing bacteria, were significantly enriched following POP intervention. These taxa are primarily responsible for the synthesis and accumulation of intestinal acetate, propionate, and butyrate, and their restoration effectively alleviated AFB_1_-induced SCFA metabolic disorders. Accumulating evidence indicates that SCFA accumulation mitigates excessive reactive oxygen species (ROS) generation and hepatic oxidative stress, suppresses inflammatory responses, and further inhibits mitochondrial-dependent hepatocellular apoptosis [3,4]. Consistent with these findings, our results demonstrated that POP treatment reversed the AFB_1_-induced reduction in cecal SCFA contents, upregulated the hepatic mRNA expression of FFAR2 and FFAR3, and attenuated oxidative stress and abnormal hepatocyte apoptosis. These improvements ultimately reduced the levels of hepatic injury markers (ALT, AST, and ALP) and efficiently ameliorated AFB_1_-triggered intestinal and hepatic pathological damage.

Although the present study demonstrates that the preventive protective effect of POP against AFB_1_-induced liver injury in rabbits is closely associated with the PI3K/AKT signaling pathway and gut microbiota, several notable experimental limitations still exist. Restricted by the large body size of experimental rabbits, in vivo injection of the PI3K-specific inhibitor LY294002 for pathway blockade assays would incur excessively high reagent costs, so such intervention verification was not performed in this work. Therefore, the current evidence supporting the involvement of the PI3K/AKT pathway in the hepatoprotective function of POP solely relies on quantitative measurements of pathway protein expression, which cannot confirm that POP alleviates AFB_1_-triggered liver injury entirely through the PI3K/AKT signaling cascade; the possibility that other parallel signaling pathways jointly mediate the protective effects cannot be excluded. On the other hand, the results related to gut microbiota and short-chain fatty acids are merely observational correlative data. This study did not establish functional verification models including fecal microbiota transplantation and antibiotic-mediated gut microbiota depletion, so we cannot directly verify that gut microbiota remodeling serves as the direct driving factor for the liver-protective activity of POP. In addition, only qualitative and quantitative HPLC analysis of monosaccharide composition and Fourier-transform infrared spectroscopy (FT-IR) characterization of basic polysaccharide properties were conducted on crude POP obtained via hot-water extraction and ethanol precipitation. Since the mixture was not further separated and purified, the homogeneous bioactive polysaccharide fraction responsible for hepatoprotective effects cannot be identified. In follow-up experiments, we will isolate and purify POP to obtain polysaccharide fractions with different molecular weights, and separately test the in vitro hepatoprotective activity and in vivo efficacy of each fraction in animal models. After screening homogeneous polysaccharides with potent bioactivity, further mechanistic validation will be carried out to provide precise structural and efficacy data to support the development of POP-based preparations.

## 5. Conclusions

In conclusion, POP pretreatment attenuates hepatic ROS accumulation, accompanied by PI3K/AKT pathway activation and suppressed mitochondrial-dependent hepatocyte apoptosis, thereby alleviating AFB_1_-induced liver injury in rabbits. This hepatoprotective effect is closely associated with restored intestinal barrier integrity, reshaped gut microbiota community structure, and elevated levels of microbial-derived short-chain fatty acids. Collectively, these findings provide a theoretical basis for employing plant-derived bioactive compounds as dietary interventions to mitigate mycotoxin-induced liver injury.

## Data Availability

The original contributions presented in the study are included in the article. Further inquiries can be directed to the corresponding authors.

## Supplementary Materials

The following supporting information can be downloaded at https://www.mdpi.com/article/10.3390/biology15151311/s1, Figure S1: HPLC detection of POP components; Figure S2: FTIR detection of POP functional groups; Figure S3: The effect of POP on normal liver tissue; Table S1: Primer seence for RT-PCR.

## Abbreviations

The following abbreviations are used in this manuscript:

AFB_1_	aflatoxin B1
POP	*portulaca oleracea* L. polysaccharide
SCFAs	short-chain fatty acids
ROS	reactive oxygen species
cyt-c	cytochrome c
PI3K	phosphatidylinositol 3-kinase
AKT	protein kinase B
AST	aspartate transaminase
ALT	alanine transaminase
ALP	alkaline phosphatase
MRM	multiple reaction monitoring
qRT-PCR	quantitative real-time polymerase chain reaction
FFARs	free fatty acid receptors
