# Supplementary material for: Portulaca oleracea L. Polysaccharide Alleviates AFB1-Induced Liver Injury in New Zealand Rabbits via the Gut–Liver Axis
Source: Biology (Basel). 2026 Aug 5;15(15):1311. doi: 10.3390/biology15151311 (PMC13465931; doi:10.3390/biology15151311)
Supplement: Supplementary file 1 [file biology-15-01311-s001.zip › biology-4429710-supplementary.pdf]

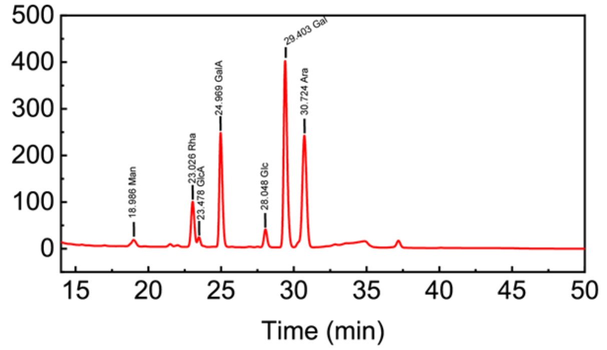

Supplementary Figure S1 HPLC detection of POP components

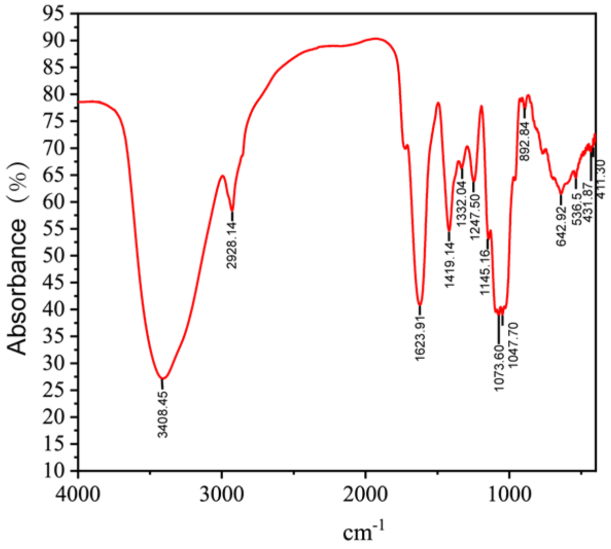

Supplementary Figure S2 FTIR detection of POP functional groups

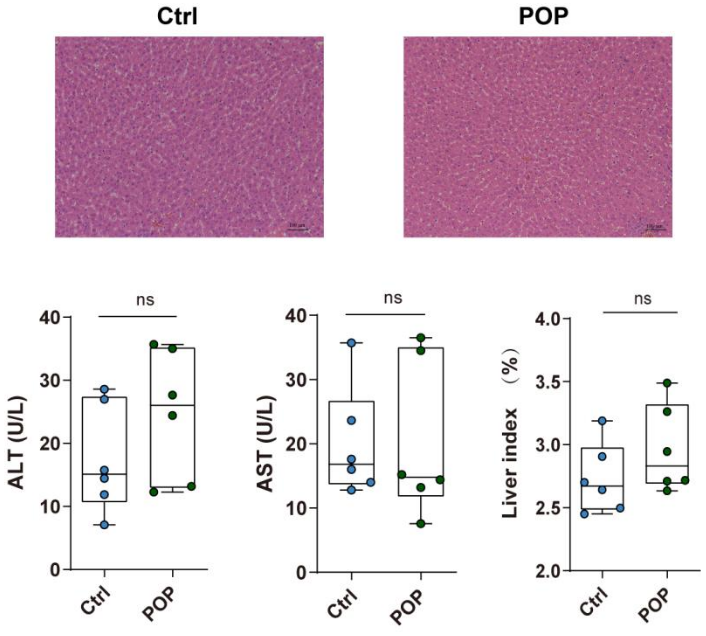

Supplementary Figure S3 The effect of POP on normal liver tissue

Supplementary Table S1. Primer seence for RT-PCR.

Gene	Primer sequence
GAPDH	Forward: 5′- GTTGTCGCCATCAATGATCCA-3′
	Reverse: 5′-TTCCCGTTCTCAGCCTTGACC-3′
IL6	Forward: 5′-AGGCACTGGCGGAAGTCAATC -3′
	Reverse: 5′-TCTCAGCAGGCAGGTCTCATTATTC- 3′
*IL1*β	Forward: 5′- GTCGTTGTGGCTCTGGAGAA-3′
	Reverse: 5′-TTGCAGAGGACGGGTTCTTC-3′
TNFα	Forward: 5′- TCCTACGTGCTCCTCACTCA-3′
	Reverse: 5′-TTCTCCAACTGGAAGACGCC-3′
Occludin	Forward: 5′- TCGTTTGAGCAGCAGCAGTA-3′
	Reverse: 5′-ACTCCCTGATCCAGTCCTCC-3′
Claudin-1	Forward: 5′- GTGCCTTGATGGTGATTGGC-3′
	Reverse: 5′-TGACAGCCATCCGCATCTTT-3′
FFAR2	Forward: 5′- TCCTTGATCCTCACGGCCTA-3′
	Reverse: 5′-ACGATCTTGAAGGGCAGCAA-3′
FFAR3	Forward: 5′- ACTTCCTCGTCTGCTTTGGG-3′
	Reverse: 5′-CAGGAGTTCAGGGTGCTGAG-3′
